# Clinical effects with customized brackets and CAD/CAM technology: a prospective controlled study

**DOI:** 10.1186/s40510-021-00386-0

**Published:** 2021-12-06

**Authors:** Julia Hegele, Lena Seitz, Cora Claussen, Uwe Baumert, Hisham Sabbagh, Andrea Wichelhaus

**Affiliations:** Department of Orthodontics and Dentofacial Orthopedics, University Hospital, LMU Munich, Goethestrasse 70, 80336 Munich, Germany

**Keywords:** CAD/CAM, Customized brackets, Orthodontic treatment, 3D treatment planning

## Abstract

**Objective:**

Nowadays, CAD/CAM technologies enrich orthodontics in several ways. While they are commonly used for diagnoses and treatment planning, they can also be applied to create individualized bracket systems. The purpose of this prospective quasi-randomized study was to evaluate the clinical efficiency of a customized bracket system and its comparison with directly bonded conventional self-ligating bracket treatment.

**Materials and methods:**

Altogether 38 patients were separated into two groups, treated either with direct bonded self-ligating brackets (Damon, Ormco, USA) or with indirect bonded customized CAD/CAM brackets (Insignia™, Ormco, USA). Overall treatment time, number of treatment appointments, number of lost or repositioned brackets, number of arch wires and wire bends, Little Irregularity Index, cephalometric analyses and ABO scores were compared. Superimpositions of the virtual set-ups and the treatment results of the CAD/CAM group were performed to evaluate the clinical realization of the treatment planning.

**Results:**

No differences between both treatment groups were found concerning overall treatment time, number of appointments and number of archwire bends. Bonding failures occurred more often using the CAD/CAM system. Indirectly bonded brackets did not have to be repositioned as often as directly bonded brackets. Treatment results with both systems were similar concerning their effects on the reduction of ABO scores. The number of used archwires was higher in the CAD/CAM group. Treatment with both systems led to further proclination of the incisors. Proclination in the lower jaw was greater than proclination in the upper jaw, and there was a statistically significant difference between the two treatment systems. Comparing the treatment results with the virtual set-ups, mesial positions were met best, followed by vertical positions. Transversal positions showed the greatest discrepancies. Concerning angles, values of angulation showed greatest accordance to the virtual set-up, while values of inclinations showed greatest discrepancies.

**Conclusion:**

In comparison with a direct bonded self-ligating bracket system the use of indirect bonded customized CAD/CAM brackets showed only minor influence on treatment efficiency and treatment outcomes. Transversal expansion, deep bite correction, expression of torque and anchorage loss remain challenges in the treatment with straight-wire appliances.

*Trial registration* DRKS, DRKS00024350. Registered 15 February 2021, https://www.drks.de/drks_web/navigate.do?navigationId=trial.HTML&TRIAL_ID=DRKS00024350.

**Supplementary Information:**

The online version contains supplementary material available at 10.1186/s40510-021-00386-0.

## Background

The introduction of intraoral scanners and CAD/CAM technology in dentistry has provided numerous innovations and possibilities. At the beginning, it was only possible to scan single teeth, and manufacturing was limited to smaller prosthetic restorations. Nowadays, the improvements in this field allow scans of larger areas, and huge amounts of data can be processed [1]. This is why these procedures are applied in almost every field of dentistry to improve the effectiveness and efficiency of dental treatments.

In orthodontics, CAD/CAM technologies are used as tools for diagnosis and treatment planning as well as for the manufacturing of aligners and fixed custom labial and lingual systems [2]. Concerning fixed appliances, this technology may facilitate the accuracy of bracket placement [3], as the position of brackets has great influence on treatment results. However, other findings show that there is no statistically significant difference in the accuracy of bracket placement between direct and indirect bonding, and neither direct nor indirect bonding can achieve ideal bracket placement [4]. To decrease errors occurring by human failure, manufacturers offer indirect bonding jigs not only to ease the bonding process, but also to evaluate the optimal bracket positions. This way, the bracket position is generated by algorithms and is no longer influenced by subjective factors like visual estimation. However, new technologies offer even more possibilities. The Insignia™ system (Ormco, Orange, USA) is a fully individualized bracket system and includes virtual set-ups to simulate the treatment results, individually manufactured bracket bases regarding tooth surface and tooth morphology with individual bracket prescriptions, individual transfer jigs and archwires.

Patients treated with a customized CAD/CAM orthodontic system showed fewer archwire appointments, shorter overall treatment time and lower American Board of Orthodontics (ABO) scores [5]. However, it is not clear whether these effects occur due to indirect bonding or due to customized brackets [6]. Additionally, current literature reports clinical outcomes only by comparing ABO scores of pre- and post-treatment records. There are no reports concerning the explicit achievement of predefined treatment goals being set by the virtual set-ups made during treatment planning. Since the advertisement of systems like Insignia™ promises a high-quality treatment by using virtual treatment planning and customized brackets, the predictability of the treatment results needed to be examined.

Therefore, the primary aim of this study was to evaluate the clinical efficiency of a directly bonded self-ligating brackets (Damon™) with an indirect bonded customized CAD/CAM bracket system (Insignia™) by comparing several clinical measurements including but not limited to ABO scores, Little Irregularity Index, treatment time, and bracket loss. In addition, the clinical realization of the treatment planning was registered by matching post-treatment scans with the virtual set-ups of patients that were treated with the Insignia™ bracket system.

## Methods

### Patient recruitment

To assess the clinical efficiency of customized brackets and CAD/CAM technology, a prospective quasi-randomized controlled study design [7] was chosen, which was approved by the local ethics committee (LMU Munich; reference 312-15) and registered (German Register of Clinical Studies; DRKS00024350). Continuous patient recruitment took place at the Orthodontic Department of the Ludwig Maximilians University, Munich, between July 2015 and June 2017. The treatment of the last patient was completed in August 2019, which also determined the end of the trial. All patients and their parental guidance gave informed consent beforehand. CONSORT 2010 flow diagram and CONSORT 2010 checklist [8] are included as Additional files 1 and 2: files 1 and 2.

To ensure good collaboration and to minimize longer waiting periods, patients suitable for inclusion were continuously enrolled following a quasi-random protocol, in which patients were alternatingly assigned to one of the study arms by the treating clinician. In the first group, treatment was done with the Insignia™ system (Ormco, Orange, USA). In the second group, patients were treated with Damon™ brackets (Ormco), which were directly bonded. Based on previous studies [5, 9], 40 patients were included. Inclusion criteria for both groups were as follows: (1) no extractions needed for treatment; (2) no orthognathic surgery necessary; (3) all permanent teeth erupted (except third molars); (4) class I malocclusion. Before the start of the treatment, initial documentation including model casts and cephalometric X-rays was done. Both groups were treated by the same orthodontist. In both groups, regular control intervals of six weeks were applied.

*Group 1* Intraoral scans of the initial situation were generated with the Lythos scanner (Ormco) and virtual set-ups were created by Insignia™. After review, modification and final approval of the virtual set-ups and the treatment plan, self-ligating brackets with individual bases, bonding jigs for indirect bonding and archwires were manufactured by Ormco and used as specified by the Insignia™ system. The archwire sequence included 0.014″ NiTi, 0.018″ NiTi, 0.016″ × 0.025″ CuNiTi, 0.016″ × 0.022″ ss, 0.019″ × 0.025″ ss and 0.019″ × 0.025″ TMA (maxilla) / 0.017″ × 0.025″ TMA (mandible) wires and was individually adjusted for each patient in form, lengths, and wire bends if needed. These individual features were given by the virtual set-ups.

Originally, this group consisted of 20 patients. Two of them withdrew their consent during treatment. Therefore, group 1 consisted of 18 patients (male: 8/18, 44.4%; female: 10/18, 55.5%) between 13.1 and 18.5 years of age.

*Group 2* Patients of the second group were treated with Damon™ brackets (Ormco), which were directly bonded. Damon standard brackets with MBT prescription and a predefined archwire sequence including 0.014″ NiTi, 0.016″ NiTi, 0.016″ × 0.022″ NiTi, 0.016″ × 0.022″ ss, 0.018″ × 0.025″ ss, and 0.019″ × 0.025″ ss were used. This group included 20 patients (male: 7/20, 35%; female: 13/20, 65%) between 12.4 and 22.2 years of age.

### Clinical measurements

At the foreseeable end of the treatment, cephalometric X-rays were repeated in both groups in order to compare the inclination of the incisors. The Little Irregularity Index [10] was determined to evaluate the amount of crowding at treatment start. The time of debonding was defined by the treating orthodontist based on the six keys of occlusion [11], overjet and overbite correction. After debonding, alginate impressions were taken. This way, ABO scores of all patients before and after the treatment were evaluated and compared by the same person. Patients’ records from both groups were analysed with respect to overall treatment time, number of treatment appointments (including emergency appointments, for example, for rebonding brackets), number of rebonded brackets (e.g. due to bracket loss or repositioning), number of arch wires, and number of wire bends additionally applied. Each of the steps “treatment”, “measurement”, and “statistics” was performed by a different author of this study independent of each other.

### Achievement of three-dimensional alignment (as planned in set-ups)

For the Insignia™ system, the achievement of the three-dimensional alignment was analysed additionally by an overlay of the virtual set-ups with the post-treatment intraoral scans for each of the patients from group 1. Virtual set-up models were exported from the Insignia™ software. Both, post-treatment scans and virtual set-ups were imported into OnyxCeph^3^™ 3D Pro (Image Instruments, Chemnitz, Germany) using the CAD exchange format STL. The post-treatment models were then superimposed onto the virtual set-up models. For the superimposition, soft tissue landmarks visible in both models were used as references, which were not affected by changes in tooth positions during treatment. Since no reliable recognizable landmarks in the soft tissue of the lower jaw (e.g. transverse folds of the mucous membrane of the palate in the upper jaw) exist, superimposition was only applied to the maxilla scans. Differences between the post-treatment tooth positions and tooth positions in the virtual set-ups were analysed for each tooth in the following dimensions: inclination, angulation, rotation, mesial (sagittal) position, buccal (transversal) position, and occlusal (vertical) position. The direction of the discrepancy was given using digit signs “+” and “−”. Absolute values (without digit signs) were used to examine how precisely the teeth met their set-up position regardless of the direction. The results were compared to the definitions of clinical acceptable ranges in all three angles and planes [9].

### Statistics

Descriptive and inferential statistical analysis was done using IBM SPSS Statistics for Mac, version 26 (IBM Corp, Armonk, NY, USA). All numerical data including cephalometric, angular and planar measurements were presented with median and range, i.e. minimum and maximum. To assess potential differences between groups, nonparametric inferential methods were applied, as most of the measurements showed deviation from the assumption of normality and due to the sample size. The Mann–Whitney U test was used to test for differences between both treatment groups and for differences between anterior and posterior teeth regarding the achievement of the 3-D alignment between virtual set-ups and post-treatment intraoral scans. To test for differences between pre- and post-treatment (U1-NL, L1-ML), the Wilcoxon signed-rank test was used. Fisher’s exact test was applied to test for a difference in proportion of gender between both treatment groups. The level of significance was set at *α* < 0.05. Post hoc power analysis was applied using G*Power (version 3.1.9.6, Mac) [12] for two-tailed tests assuming *α* = 0.05 and a power of 0.8. Additionally, based on this assumptions a sensitivity analysis was carried out based on the anticipated sample size (*N* = 40; *N*_1_ = *N*_2_ = 20), resulting in a minimum detectable effect size of *d* = 0.931. Due to the drop-outs in group 1 (*N* = 38; *N*_1_ = 18, *N*_2_ = 20), the minimum detectable effect size increased to *d* = 0.958. The results of the power analysis were reported (Cohen’s *d*, power and correlation if appropriate) and considered in the interpretation.

## Results

The first patient group was treated with the Insignia™ system and consisted of 18 patients (male: 8/18, 44.4%; female: 10/18, 55.5%) between 13.1 and 18.5 years of age (Table 1). The second group, treated with Damon™ brackets, included 20 patients (male: 7/20, 35%; female: 13/20, 65%) between 12.4 and 22.2 years of age (Table 1). Both groups did not differ in the proportion of male and female patients (Fisher’s exact test; *p* = 0.741).

**Table 1:** Descriptive statistics of conditions during therapy including ABO scores

	Total	Group 1	Group 2	*p* Values (1–2)	Effect size
Patients [*n* (%)]	38 (100)	18 (47.4)	20 (52.6)		
Sex [*n* (% of total)]					
Male	15 (39.5)	8 (21.1)	7 (18.4)	0.741^a^	0.097^c^
Female	23 (60.5)	10 (26.3)	13 (34.2)		
Age at the start of treatment (years)	14.3 [12.4; 22.2]	14.3 [13.1; 18.5]	14.3 [12.4; 22.2]	0.696^b^	0.044^d^
Little Irregularity Index	3.6 [0.5; 13.0]	4.7 [0.5; 8.0]	2.7 [1.6; 13.0]	0.051^b^	0.438^d^
Treatment time (months)	16.7 [10.1; 35.2]	16.7 [13.0; 30.1]	16.8 [10.1; 35.26]	0.654^b^	0.084^d^
Number of brackets lost	1.0 [0; 19]	2.0 [0; 19]	1 [0; 3]	0.035^b^	0.813^d^
Number of brackets repositioned	0 [0; 10]	0 [0; 5]	0.5 [0; 10]	0.024^b^	0.611^d^
Number of archwires	4.5 [3; 6]	6 [3; 6]	4 [3; 6]	< 0.001^b^	1.3^d^
Number of archwire bends	2.5 [0; 14]	2 [0; 5]	5 [0; 14]	0.093^b^	0.789^d^
Number of appointments	15.0 [8; 28]	16.5 [10; 28]	14 [8; 25]	0.082^b^	0.494^d^
ABO scores before treatment	46 [30; 62]	46 [30; 62]	48 [32; 62]	0.874^b^	0.105^d^
ABO scores after treatment	13 [3; 24]	12 [3; 23]	16 [7; 24]	0.133^b^	0.442^d^
ABO score change within treatment	32 [16; 53]	32 [20; 53]	32 [16; 48]	0.806^b^	0.248^d^

If not otherwise stated, median and range [minimum; maximum] were reported including effect sizes. The Mann–Whitney U test was applied to compare both groups: group 1 (individualized CAD/CAM system) and group 2 (conventional self-ligating system)

^a^Fisher’s exact test, ^b^exact significance Mann–Whitney U test, ^c^Cohen’s *w*, ^d^Cohen’s *d*

**Fig. 1:**
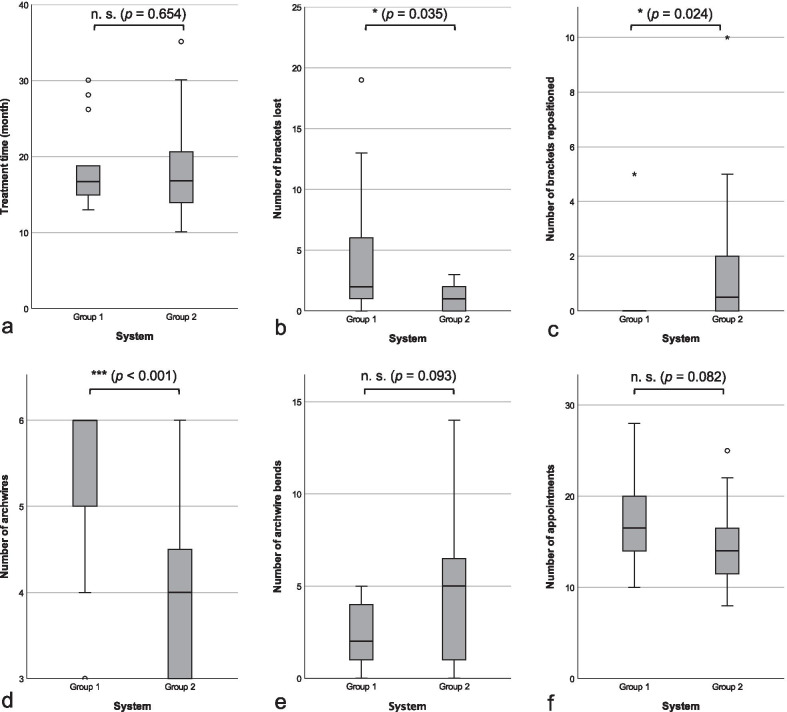
Distribution of treatment time (**a**), and the number of brackets lost (**b**), brackets repositioned (**c**), archwires used (**d**), archwire bends (**e**), and appointments (**f**) in groups 1 and 2. Pairwise comparisons were done using the Mann–Whitney U test and their test statistics (n.s., not significant; *, *p* < 0.05; ***, *p* < 0.001)

The median overall treatment time of the patient cohort was 16.7 months (range 10.1–35.2; group 1: median 16.7 months; group 2: median 16.8 months; Table 1). This difference was statistically not significant (*p* = 0.654) (Table 1, Fig. 1a).

### Little Irregularity Index

Little Irregularity Indices of both groups before the treatment showed no statistically significant difference (*p* = 0.051; Table 1), indicating a similar amount of crowding in both groups.

### Effectiveness

The number of appointments did not differ statistically significant between both groups (*p* = 0.082) (Table 1, Fig. 1f). Archwires were changed significantly more frequently in group 1 than in group 2 (*p* < 0.001) (*d* = 1.3, power = 0.967) (Table 1, Fig. 1d); the number of wire bends showed no statistically significant difference (*p* = 0.093) (Table 1, Fig. 1e). In addition, bracketloss was observed more often in group 1 than in group 2 (*p* = 0.035; *d* = 0.813, power = 0.661) (Table 1, Fig. 1b), whereas brackets were replaced more frequently in group 2 than in group 1 (*p* = 0.024; *d* = 0.611, power = 0.432) (Table 1, Fig. 1c).

### ABO scores

The ABO scores before treatment did not differ significantly between both study groups (*p* = 0.874). After treatment, the ABO scores in both groups were reduced, but did not significantly differ between both groups (*p* = 0.806) (Table 1).

### Cephalometric analyses

Concerning cephalometric analyses, especially the values of the inclination of the incisors and their development during the treatment, seemed particularly interesting.

In both groups, upper incisors (U1-NL) already showed protrusion before the treatment (group 1: median 111.4°; group 2: median 113.3°; Table 2). The protrusion of the upper incisors increased statistically significant in group 1 after treatment (median: 114.0; *p* = 0.008; *d* = 0.654, *r* = 0.857, power = 0.970). In group 2, the inclination of the upper incisors did not change significantly during treatment (*p* = 0.191). However, these changes were not statistically significant different between both groups (*p* = 0.206) (Table 2).

**Table 2:** Descriptive statistics of U1-NL and L1-ML values before and after the treatment for both groups, group 1 (individualized CAD/CAM system) and group 2 (conventional self-ligating system)

Measurement	Group	Before treatment (Median [range])	After treatment (Median [range])	*P* values Wilcoxon test (effect size)	Absolute difference (Median [range])	*p* Values U test (effect size)
U1-NL (°)	Group 1	111.4 [98.0; 126.6]	115.2 [101.6; 125.2]	0.008^a^ (0.654)	3.2 [0.7; 10.8]	0.206^b^ (0.085)
	Group 2	114.5 [97.3; 129.3]	114.7 [98.8; 127.2]	0.191^a^ (0.654)	5.5 [0.3; 16.5]	
L1-ML (°)	Group 1	96.4 [87.7; 105.8]	99.4 [87.8; 107.3]	0.006^a^ (0.721)	4.4 [0.3; 13.9]	0.016^b^ (0.866)
	Group 2	94.5 [82.5; 106.8]	101.5 [90.7; 116.7]	< 0.001^a^ (1.584)	8.5 [2.1; 18.7]	

Matched pairs (before vs. after treatment, B-A) were compared with the Wilcoxon signed-rank test, and pairwise comparisons of the measurements between group 1 and group 2 were analysed using Mann–Whitney U test, and *p* values and effect sizes (Cohen’s *d*) reported

^a^Asymptotic significance, ^b^exact significance

In both groups, the lower incisors (L1-ML) showed protrusion before treatment (group 1: median 96.4°; group 2: median 94.5°). In both groups, treatment led to statistically significant changes of inclination in terms of further protrusion (group 1: *p* = 0.006, *d* = 0.721, *r* = 0.653, power = 0.983; group 2: *p* < 0.001, *d* = 1.584, *r* = 0.776, power > 0.999). Lower incisors of group 2 were statistically significant more protruded than lower incisors of group 1 (*d* = 0.866, power = 0.717; Table 2).

### Achievement of teeth positions (as planned in set-ups)

Concerning achieving the predefined teeth positions, the results of this study showed that sagittal positions were met best, while transversal positions showed the greatest discrepancies to the virtual set-up positions (Table 3).

**Table 3:** CAD/CAM group analysis with reference to the achievement of teeth positions and their three-dimensional alignment as planned in the set-ups for the maxilla

	All teeth (*n* = 252)	Anterior teeth (*n* = 108)	Posterior teeth (*n* = 144)	Effect size (Cohen’s *d*)	U test (A-P)	
					*p* Values	Sig. level^a^
Absolute inclination (°)	4.30 [0; 18.40]	4.30 [0; 17.30]	4.25 [0; 18.40]	0.002	0.420	N.s
Absolute angulation (°)	2.80 [0; 42.00]	2.20 [0; 42.00]	3.20 [0.10; 31.00]	0.143	0.020	*
Absolute rotation (°)	3.75 [0; 32.70]	4.65 [0; 23.60]	3.20 [0.20; 32.70]	0.271	0.017	*
Absolute mesial movement (mm)	0.51 [0; 7.34]	0.50 [0; 2.09]	0.51 [0; 7.34]	0.193	0.357	N.s
Absolute vestibular movement (mm)	0.84 [0; 5.07]	0.70 [0.01; 2.23]	1.02 [0; 5.07]	0.544	0.001	***
Absolute occlusal movement (mm)	0.63 [0.01; 5.19]	0.57 [0.01; 5.19]	0.68 [0.01; 3.21]	0.153	0.065	N.s

Absolute values of discrepancies (median [min; max]) of the dental arch for all teeth and for both frontal and posterior teeth separately and their effect size (Cohen’s *d*) were reported. Given are inclination, angulation, rotation, mesial (sagittal) position, vestibular (transversal) position and occlusal (vertical) position. Mann–Whitney U test was applied to compare anterior and posterior teeth

^a^*, *p* < 0.05; ***, *p* ≤ 0.001; *n.s.* not significant

**Fig. 2:**
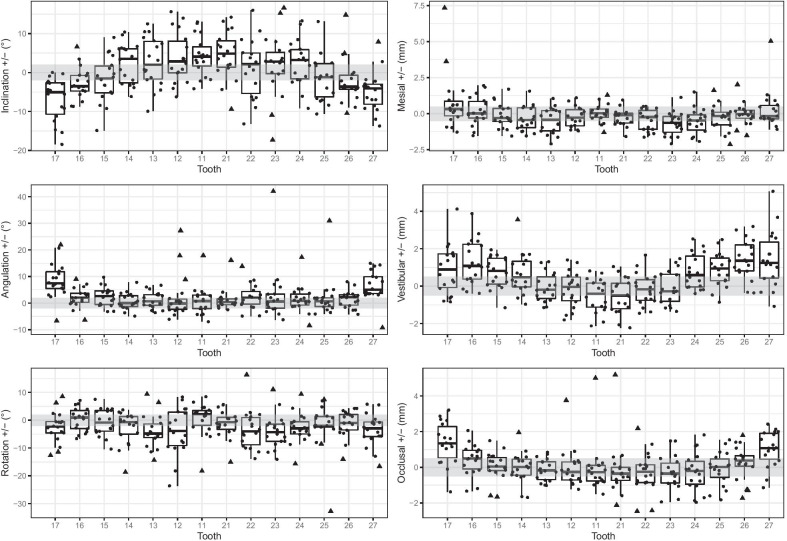
Values of discrepancies of each tooth in the all three angles (inclination, angulation, rotation) and all three planes (mesial [sagittal], vestibular [transversal], occlusal [vertical]) between set-up and the situation after treatment. The grey rectangles define the “allowed” or “tolerable” areas according to Larson et al. [9]: ± 2.0° for angular and ± 0.5 mm for linear measurements

Regarding vestibular/transversal positions, the anterior teeth showed smaller discrepancies than posterior teeth (Table 3). Only the positions of the lateral incisors and the canines were achieved within a clinically acceptable range of 0.5 mm, whereas other teeth showed larger discrepancies. Both tooth segments (anterior tooth segment and posterior tooth segment) differed statistically significant from the median of the allowed range of 0–0.5 mm (anterior teeth *p* < 0.001, posterior teeth *p* < 0.001). Furthermore, the results showed poorest positions for the molars. Concerning the direction of the discrepancies, the transversal position of the posterior teeth was too palatal, while the transversal position of the anterior teeth was too buccal/anterior (Fig. 2).

The planned sagittal/mesial movements in our study were attainable with discrepancies lower than 0.5 mm for all teeth except upper left canines and performed better for anterior teeth than for posterior teeth (Table 3). Concerning the direction of discrepancies, all teeth showed the tendency to be positioned too far anterior, except upper right second molars that were positioned too far posterior (Fig. 2).

In the vertical/occlusal plane, discrepancies for anterior teeth in our study were lower than for posterior teeth (Table 3). They were most precise for second premolars, whereas the vertical position for the second molars showed worst results with discrepancies higher than 0.5 mm. Considering the directions, anterior teeth tended to show suprapositions compared to the set-ups. Upper posterior right and left teeth (15–17, 25–27) were not extruded enough (Fig. 2).

### Achievement of three-dimensional alignment (as planned in set-ups)

The results of our study showed in terms of reaching all three predefined angles, that the values for angulation showed greatest accordance with the virtual set-up, while values of inclinations showed greatest discrepancies (Table 3).

The inclination of the virtual set-up positions was met more precisely by posterior teeth than by anterior teeth. In our study, the planned movement in this dimension was achieved in a clinically acceptable range of 2° by second premolars. Worst results were shown in the movement of second molars and upper left central incisors. In comparison with the virtual set-up, inclination values of upper frontal teeth including the first premolars (14–24) were too high, whereas for the upper posterior right and left teeth (15–17, 25–27) these were too low (Fig. 2).

Regarding the values for angulation, posterior teeth showed greater discrepancies than anterior teeth (Table 3). Only molars showed discrepancies greater than 2° as well as the greatest range of values. Lowest discrepancies could be shown by canines and first premolars. Except of the upper right lateral incisors, the angulation of all teeth was too mesial (Fig. 2).

The results of our study showed that derotation tends to be performed better by posterior teeth than by anterior teeth (*d* = 0.271, power = 0.543; Table 3). Upper left central incisors, right first premolars, second premolars on both sides and first molars on both sides showed discrepancies lower than 2°, whereas the position of the canines after the treatment showed the greatest discrepancies to their position in the virtual set-up. Except upper right central incisors, right second premolars and right first molars, the rotation of all teeth was too distal in the end of the treatment (Fig. 2).

## Discussion

Since new CAD/CAM technologies offer new possibilities concerning fixed appliances in orthodontics, their clinical effectiveness and efficiency needed to be evaluated. Our study compared various treatment variables of directly bonded customized brackets with individualized CAD/CAM brackets from the same manufacturer that were indirectly bonded. Furthermore, the treatment results of the latter system were evaluated by superimposing these with the virtual set-ups.

Inclusion criteria of our study kept the sample size relatively small and recruited patients with rather simple cases. This way, the treatment was completed in a measurable period of time and consistent material application for each patient could be guaranteed. Additionally, both treatment modalities were applied by the same orthodontist. Thus, differences in both, the concept of the ideal virtual set-up and the debonding criteria, were avoided if applied by different orthodontists.

In comparison with earlier studies, we did not find a statistical significant difference in treatment time between both study groups [5, 6]. However, our study was different in terms of design, order and inclusion criteria. While earlier data were collected in retrospective trials with different timing and several orthodontists involved in treatment, our study was performed as a prospective trial and in a defined chronological order. In addition, there were no standardized intervals between the appointments in earlier studies, whereas the patients in our study were scheduled with appointments ~ 6 weeks to avoid an impact of variable appointment intervals on total treatment time [5]. Although emergency appointments were recorded as well, this might explain the similar numbers of appointments of both groups in our study.

We observed that brackets were lost more often in group 1, which included indirectly bonded CAD/CAM brackets. However, though this was a large-sized effect (*d* = 0.813), it was missing the necessary power (power = 0.661). Therefore, these findings should be considered with caution, especially, since earlier reports showed that there were no differences in terms of bonding failure rates between directly and indirectly bonded custom brackets [4]. This leads to the assumption that clinicians might need to develop some routine in the process of indirect bonding. Since rebonding of previously lost brackets sometimes requires to return to the previous wire dimension, the number of wires used in our study was subsequently higher in group 1 than in group 2. Nevertheless, the use of bonding jigs by Insignia™ might lead to a reduction in bracket reposition. Being a medium-sized effect (*d* = 0.611), the necessary power was missing (power = 0.432). Even though this fact implies that bracket placement was more accurate for indirectly bonded brackets, this assumption needs to be viewed with caution, because additional wire bends for accurate levelling were needed equally often in both groups. An orthodontist with little experience would benefit in the initial phase of treatment from the computer-assisted method: digital placement and indirect bonding as well as prefabricated archwires do not require higher manual skills. Nevertheless, because of the individual biological reaction of each patient, good manual skills and clinical experience are necessary to finish the case.

Concerning ABO scores, our study confirmed earlier findings that showed no differences between directly bonded custom brackets, indirectly bonded custom brackets, and indirectly bonded CAD/CAM brackets [6]. Furthermore, both patient groups showed similar scores before the treatment emphasizing that the degree of severity of both groups was equivalent and reaffirms the comparability of the treatment groups. The ABO score does not take into account the sagittal position change of the incisors of the respective jaws. Any compensatory movements of the front therefore could not be evaluated this way. This is why also cephalometric analyses were necessary.

Cephalometric analyses showed that incisors were protruded in both groups before treatment and that during treatment incisors proclined even more. Using a fixed buccal bracket system, load application cannot be placed in the centre of resistance. Biomechanically, treatment with straight-wire systems leads to further protrusion of frontal teeth. Our results confirm earlier findings that showed proclination of mandibular incisors using brackets of the Damon system [13]. Even though the amount of crowding in the beginning of the treatment was similar in both groups, mandibular incisors in group 1 showed less protrusion during the treatment than in group 2. Though being a large-sized effect (*d* = 0.886), its statistical power of 0.717 was just below the limit. Nevertheless, this could be an effect of individual bracket bases and individual torque values of the brackets in group 1. To verify this finding, a superimposition of the lower jaws would have been beneficial. For superimposition of virtual set-ups with the corresponding scans of the treatment results, only data of the maxilla were used leading to a smaller data set. However, the method of superimposing landmarks of the palate was used in several studies before and is considered the most accurate superimposition approach applied to teeth of the maxilla [14]. So far, no valid method for superimposition of intraoral scans exists. Usage of CBCT data might have been a more accurate way, since this allows the registration of mandibular tooth movements. But generating CBCTs for patients that fit our inclusion criteria would have been ethically not acceptable [14].

Our superimposition data described the differences between virtual set-up and treatment results averaged over anterior and posterior teeth, respectively. Discrepancies between the virtual set-up and the clinical result of the treatment do not necessarily mean that the clinical result itself is not acceptable. It only shows that the planned tooth movement could not be performed as it was supposed. Our superimposition results showed that positions of anterior teeth were met better than the planned positions of posterior teeth. This might be caused by the smaller root surface and therefore less anchorage of anterior teeth.

In our study, tooth positions showed greatest discrepancies in the vestibular/transversal plane. The results showed that the posterior teeth have not reached their planned transversal positions and remained too palatal. Dental crowding in non-extraction cases is normally dissolved by transverse expansion and proclination of the incisors [15]. Instead of performing physical movements to widen the dental arches, posterior teeth were rather tipped buccally. This effect can be explained by the mechanical limitation of a fixed bracket appliance. It was shown that transversal expansion of the arch by tipping of teeth will lead to relapse [16], especially in the upper molar region [17].

Since transversal tooth movement could not be performed as planned, space gain was created by anterior positioning of the frontal teeth. The dental arch of the maxilla can best be compared with the geometrical figure of an ellipse. The perimeter “*P*” of the ellipsoid is calculated by the Ramanujan approximation for the circumference of an ellipse and is given by the formula [18]:

$$P = \pi \left( {a + b} \right)\left\{ {1 + \frac{3h}{{10 + \sqrt {\left( {4 - 3h} \right)} }}} \right\},\quad {\text{where}}\;h = \frac{{\left( {a - b} \right)^{2} }}{{\left( {a + b} \right)^{2} }}.$$

This formula shows that a reduction of the ellipse’s width “*a*” while the perimeter of the arch “*P*” remains constant will lead to an expansion of the height “*b*” of that ellipse. Knowing that transversal movements cannot be performed as planned, alternative strategies for the creation of space must be developed before the treatment. One possibility might be an overcorrection in the virtual set-up. However, this might lead to even more buccal tipping of the molars as presented in our results. Another possibility would be to create space by precisely planned slicing in stages.

Our inclination data showed that upper incisors have not reached the required torque levels simulated in the virtual set-up. This was an expected effect, since even with arch dimensions of 0.018″ × 0.025″ and 0.019″ × 0.025″ stainless steel, no effective torque can be transmitted with a right angular slot geometry [19–24]. Clinical studies also confirm that even different prescriptions and torque angles do not result in different axis positions [25]. In addition, especially with passive self-ligating brackets, the play between slot and archwire is larger in comparison with conventional brackets due to a larger slot dimension [23]. Variations in the fabrication accuracies of the slot and the archwire dimension as well as biological factors lead to insufficient torque transmission [26].

Concerning the achieved tooth positions, mesial/sagittal positions were met best and discrepancies were in a clinical acceptable range of less than 0.5 mm. Regarding the direction of the discrepancies, our results showed that teeth were positioned too mesially. The cause of this might have been the use of low frictional brackets without auxiliary devices or selectively placed ligatures to prevent mesial drifts of the teeth and to keep anchorage levels high. It has been shown that lower incisors of patients treated with the Damon system were significantly advanced and proclined [13]. However, this effect was also shown in the control group that was treated with edgewise brackets [13]. This leads to the assumption that the straight-wire appliance itself might be responsible for the observed proclination due to biomechanical side effects. Therefore, the proclination and advancement of the incisors should be considered in treatment planning with a straight-wire appliance regardless of the used system and even if individual CAD/CAM brackets are used. This was confirmed by the angulation values that showed mesial-tipping of teeth, which can be seen as another indication of anchorage loss. In summary, the application of a CAD/CAM system requires an exact staging and selectively placed ligatures.

Values of rotation angles showed that canine positions differed the most, which can be related to their larger root surface. The clinically relevant deviation of more than 2° in ~ 50% of the teeth showed that the application of passive self-ligating brackets and archwires of small dimensions not necessarily result in an accurate derotation even if individual bracket bases were used. Passive self-ligating brackets show worse results concerning rotational control than active self-ligating brackets [27], and the used ligation technique has great influence on rotational control [28]. Taken together, we concluded that additional ligatures were needed even with individual bracket bases.

Besides accurate levelling, another main task of fixed buccal appliances is the correction of deep bite including levelling of the curve of Spee. In the upper jaw, this would lead to a relative intrusion of incisors and a relative extrusion of posterior teeth. According to the literature, levelling the curve of Spee with straight-wire appliances predominantly leads to molar extrusion and only slight intrusion of incisors occurs [29] since biological and biomechanical factors limit the intrusion movement [30]. As such, the Insignia™ bracket system is unlikely to perform incisor intrusion as planned in the virtual set-ups in the first place when straight-wire techniques are used. This is also shown by the vertical discrepancies of incisor positions between the virtual set-ups and the treatment results. Furthermore, the results of our study showed that posterior teeth failed to perform extrusion as planned. As a result, deep bite correction could not be performed as planned in the virtual set-ups, and the use of individual bracket bases cannot correct this type of malocclusion on its own.

Randomized clinical trials (RCTs) are considered being of highest evidence. Nevertheless, it should be mentioned that even RCT studies are not necessarily bias-free [31]. Herein, a single-centre study was described applying a quasi-randomized protocol for patient allocation. As such waiting times for patients were limited, and all patients were treated in a reasonable period of time. As discussed by Bondemark and Ruf [31], complete blinding in a clinical study like this is almost impossible, since patient and caregiver exactly know which treatment modality was applied. However, treatment, measurements, and statistics were performed by different persons, which also reduced the risk of bias. Therefore, when weighting and interpreting the results, these points should be taken into account.

## Conclusions

1. Virtual treatment planning and individualized bracket bases and bracket positioning seemed to have no influence on overall treatment time, number of appointments, number of archwire bends, and the reduction of ABO-scores.
2. Treatment with both systems leads to further proclination of incisors.
3. Comparing the treatment results with the virtual set-ups, mesial positions were met best, followed by vertical positions. Transversal positions showed the greatest discrepancies. Concerning angles, values for angulation showed greatest accordance to the virtual set-up, while values of inclinations showed greatest discrepancies.
4. The use of individualized bracket systems and the development of virtual set-ups does require well-considered space management as well as exact planning of anchorage devices and selectively placed ligatures.
5. Transversal expansion, deep bite correction, expression of torque, and anchorage loss remain challenges in the treatment with straight-wire appliances.

## Supplementary Information

**Additional file 1.** CONSORT 2010 flow diagram.

**Additional file 2.** CONSORT 2010 checklist.

## Data Availability

The datasets used and/or analysed during the current study are available from the corresponding author upon reasonable request.

## Abbreviations

ABO: American Board of Orthodontics
CAD/CAM: Computer-aided design/computer-aided manufacturing
SD: Standard deviation
