# Supplementary figures and images for: Clinical effects with customized brackets and CAD/CAM technology: a prospective controlled study
Source: Prog Orthod. 2021 Dec 6;22:40. doi: 10.1186/s40510-021-00386-0 (PMC8645527; doi:10.1186/s40510-021-00386-0)

## CONSORT 2010 Flow Diagram

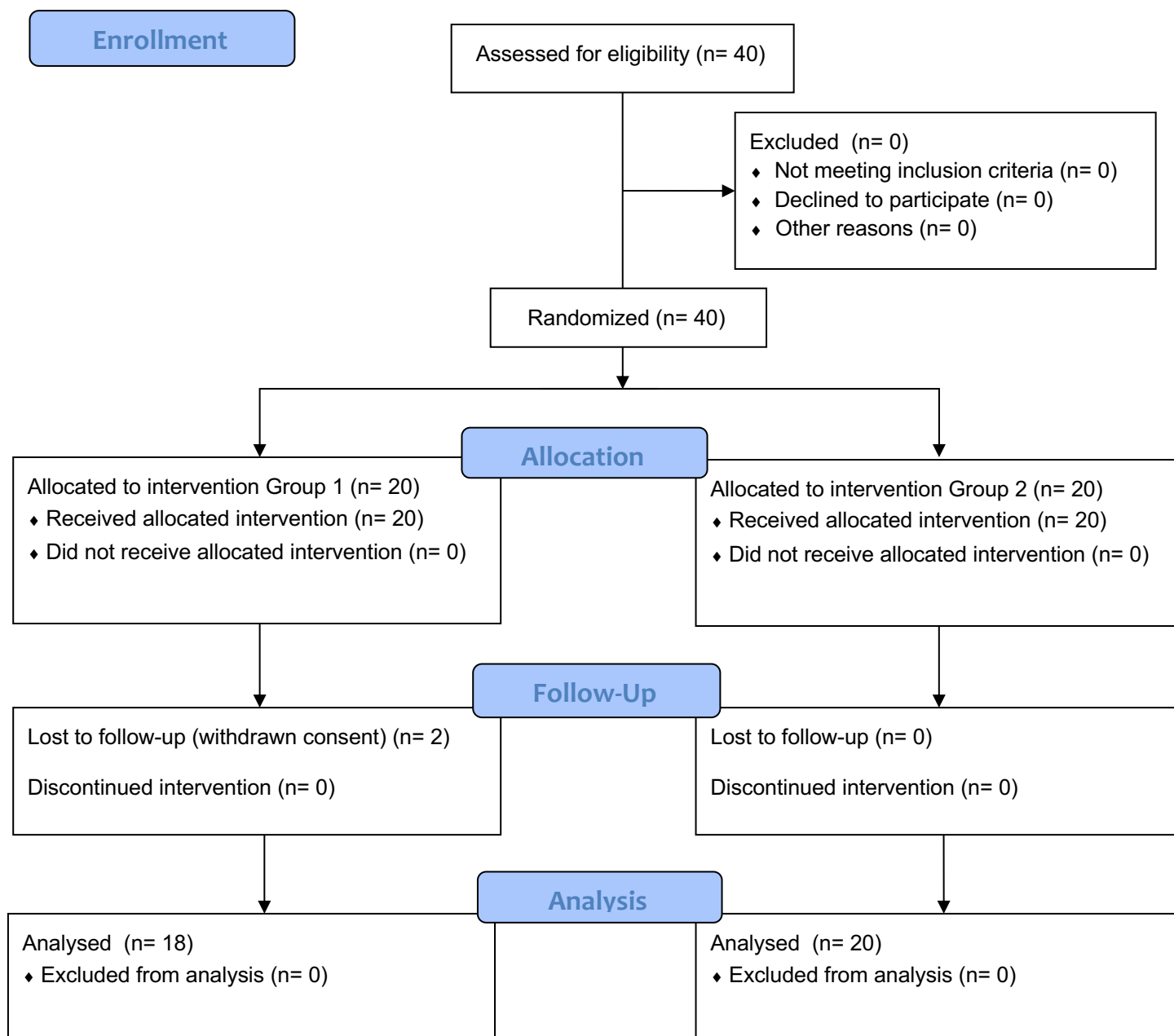

Supplement: Supplementary file 1 — Additional file 1. CONSORT 2010 flow diagram. [file 40510_2021_386_MOESM1_ESM.pdf]
